# IL-37 Targets TSLP-Primed Basophils to Alleviate Atopic Dermatitis

**DOI:** 10.3390/ijms22147393

**Published:** 2021-07-09

**Authors:** Tianheng Hou, Miranda Sin-Man Tsang, Lea Ling-Yu Kan, Peiting Li, Ida Miu-Ting Chu, Christopher Wai-Kei Lam, Chun-Kwok Wong

**Affiliations:** 1 Department of Chemical Pathology, The Chinese University of Hong Kong, Prince of Wales Hospital, Hong Kong, China; houth@link.cuhk.edu.hk (T.H.); sinmantsang@cuhk.edu.hk (M.S.-M.T.); idachu@cuhk.edu.hk (I.M.-T.C.); 2 State Key Laboratory of Research on Bioactivities and Clinical Applications of Medicinal Plants, Institute of Chinese Medicine, The Chinese University of Hong Kong, Hong Kong, China; 1155122453@link.cuhk.edu.hk (L.L.-Y.K.); peiting@link.cuhk.edu.hk (P.L.); 3 Faculty of Medicine and State Key Laboratory of Quality Research in Chinese Medicines, Macau University of Science and Technology, Macao, China; wklam@must.edu.mo

**Keywords:** allergy, atopic dermatitis, basophils, cytokines, IL-37, inflammation

## Abstract

Atopic dermatitis (AD) represents a severe global burden on physical, physiological and mental health. Innate immune cell basophils are essential for provoking allergic inflammation in AD. However, the roles of novel immunoregulatory cytokine IL-37 in basophils remain elusive. We employed in vitro co-culture of human basophils and human keratinocyte HaCaT cells and an in vivo MC903-induced AD murine model to investigate the anti-inflammatory mechanism of IL-37. In the in vitro model, IL-37b significantly decreased Der p1-induced thymic stromal lymphopoietin (TSLP) overexpression in HaCaT cells and decreased the expression of TSLP receptor as well as basophil activation marker CD203c on basophils. IL-37 could also reduce Th2 cytokine IL-4 release from TSLP-primed basophils ex vivo. In the in vivo model, alternative depletion of basophils ameliorated AD symptoms and significantly lowered the Th2 cell and eosinophil populations in the ear and spleen of the mice. Blocking TSLP alleviated the AD-like symptoms and reduced the infiltration of basophils in the spleen. In CRISPR/Cas9 human IL-37b knock-in mice or mice with direct treatment by human IL-37b antibody, AD symptoms including ear swelling and itching were significantly alleviated upon MC903 challenge. Notably, IL-37b presence significantly reduced the basophil infiltration in ear lesions. In summary, IL-37b could regulate the TSLP-mediated activation of basophils and reduce the release of IL-4. The results, therefore, suggest that IL-37 may target TSLP-primed basophils to alleviate AD.

## 1. Introduction

Atopic dermatitis (AD), also known as atopic eczema, is a common chronic or recurrent inflammatory skin disease affecting 15–20% of children [1] and 1–3% of adults worldwide. It is characterized by eczema and pruritus of dry skin [2]. The two main pathological features of AD are abnormalities in the structure and function of the epidermis and skin cutaneous inflammation [2]. The damage-associated molecular pattern molecules, including alarmins such as IL-1β, IL-25, IL-33 and thymic stromal lymphopoietin (TSLP), are released in response to tissue damage, for inducing inflammation primed by the percutaneous allergen [3]. In the lesional area, the affected skin shows inflammatory cell infiltration, such as basophils, eosinophils and particularly CD4+ T helper (Th) cells [2,4]. Non-lesional skins also show subclinical inflammation with an increased population of Th2 cells, Th22 cells and pro-inflammatory cytokine milieu [5]. Activated Th2 cells can facilitate IgE class switching and the production of antigen-specific IgE. When encountering the same antigen, the Fc region of the IgE–antigen complex would bind to the high-affinity receptor (FcεRI) on mast cells and basophils, leading to the release of histamine, a pruritogen. Basophils can cause a skewed Th2 reaction by processing haptens and peptide antigens alone but need to cooperate with dendritic cells (DCs) to process protein antigens to induce a Th2 response [6,7].

TSLP–TSLP receptor signaling plays a critical role in AD-like inflammation [8,9]. In the presence or absence of IL-3, TSLP can foster the development and optimal response of basophils [9]. In our previous study, TSLP was found to induce chemotactic and pro-survival effects in eosinophils [10]. TSLP-primed basophils are involved in the pathogenesis of eosinophilic esophagitis, a food allergy-associated inflammatory disease [11]. A recent placebo-controlled clinical phase III trial has shown that patients receiving anti-TSLP monoclonal antibody had improved clinical symptoms compared to those receiving a placebo in uncontrolled asthma [12]. TSLP has been shown to play a vital role in the pathogenesis of AD, which takes part in the early initiation of Th2 immune responses [13,14]. In addition, TSLP could induce the maturity of a functionally distinct basophil subgroup in an IgE-independent manner. TSLP-elicited basophils would release IL-4, which primes the Th2 response [8]. Patients with AD have antigen-specific IgE-activated basophils [15], consistent with basophils acting in an IgE-dependent manner in the pathogenesis of chronic murine AD [16]. Of note, the depletion of basophils significantly reduces the infiltration of eosinophils and neutrophils, together with skin thickness [16].

IL-1 family cytokine IL-37/IL-1F7, a fundamental inhibitor of immunity, can downregulate the Th2 immune response and inflammation by suppressing the production of pro-inflammatory mediators and cytokines in innate and adaptive immunity [17]. Among the five different IL-37 splice variants, IL-37b is the most effective and best-characterized variant [18]. As no similar IL-37 gene has been discovered or characterized in murine species, we herein investigate the anti-inflammatory mechanisms of IL-37 in AD by using recombinant human IL-37b and CRISPR/Cas9 human IL-37b knock-in mice. As a dual-function cytokine, IL-37 can exert robust anti-inflammatory effects through extracellular receptor-mediated signaling pathways and intracellular nuclear transcription mechanisms. Extracellularly, tripartite ligand-receptor complex IL-37–IL-1R8–IL-18Rα is responsible for the transduction of anti-inflammatory signals. Intracellular IL-37 interacts and combines with Smad3; then, it translocates to the nucleus, whereas it inhibits the transcription of pro-inflammatory genes [19]. IL-37 can regulate systemic/local inflammation and skewed Th2 cytokines/chemokines by suppressing the production of pro-inflammatory mediators [20]. However, whether IL-37b regulates TSLP-mediated basophils to control the onset and progress of AD remains elusive. Therefore, we used in vitro, in vivo and ex vivo experiments to elucidate the underlying anti-inflammatory mechanism of IL-37, focusing on its regulatory effect on basophils, in AD.

## 2. Results

### 2.1. The Effect of IL-37b on TSLP Expression and Activation of Basophils

We observed a significant increase in IL-37b, TSLP and TNF-α but a significant decrease in Smad3 transcription (Figure S1a,d–f) in human HaCaT cells upon Der p1 stimulation. IL-37b treatment could, however, reverse the downregulation of Smad3, as well as reverse the upregulation of TSLP and TNF-α transcription induced by Der p1, as supported by qPCR and Western blot (Figure S1g–j). Interestingly, there was no difference in the transcription of IL-37 receptor IL-18Rα and IL-1R8 in HaCaT cells upon Der p1 stimulation (Figure S1b,c). The results indicate that Der p1 could induce inflammation through inhibiting the IL-37 intracellular signaling pathway but not the extracellular signaling pathway (Figure S1b–d).

**Figure 1:**
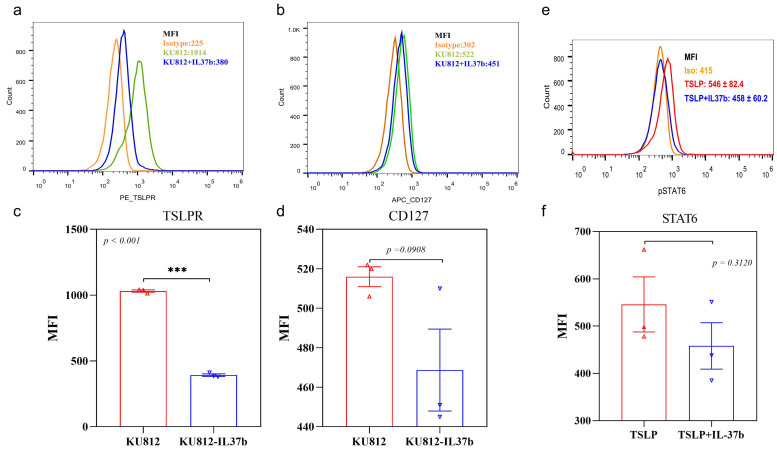
Effects of IL-37b on the expression of TSLP receptor of KU812 cells analyzed by flow cytometry. (**a**) TSLPR and (**b**) CD127; (**c**,**d**) quantitative flow cytometry analysis of TSLPR and CD127; (**e**) pSTAT6 and (**f**) quantitative analysis of pSTAT6. Bar charts are shown as mean ± SEM of triplicate independent experiments. *** *p* < 0.001 when compared between the groups.

Since TSLP can prime basophils through basophil receptors TSLPR and CD127, we examined the in vitro effect of IL-37b on the expression of TSLP receptors on basophils. We found that IL-37b could suppress the expression of TSLPR and CD127 on KU812 cells (Figure 1a–d). KU812 is a basophilic cell line model that was adopted in our previous mechanistic study for allergic disease [21]. We observed that IL-37b could reduce the TSLP-induced phosphorylation of intracellular STAT6 (Figure 1e,f), thereby indicating that IL-37b might suppress the expression of TSLP receptors by downregulating p-STAT6.

**Figure 2:**
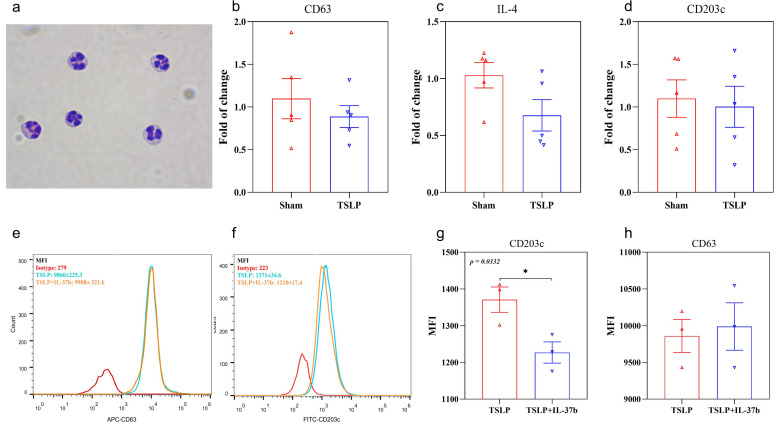
Effects of IL-37b on the expression of basophil activation markers and IL-4 by qPCR. (**a**) Basophils isolated from human PBMC; (**b**–**d**) qPCR quantitative analysis of CD63, IL-4 and CD203c; (**e**,**f**) flow cytometry analysis of CD63 and CD203c; (**g**,**h**) quantitative flow cytometry analysis of CD63 and CD203c. Bar charts are shown as mean ± SEM of *n* = 3 or 5. * *p* < 0.05 when compared between the groups.

Furthermore, primary human basophils isolated from PBMC (Figure 2a) were stimulated by TSLP. Although no significant difference was found in the transcription level of IL-4 and basophil activation marker CD63 and CD203c upon TSLP stimulation (Figure 2c,d, all *p* > 0.05), our flow cytometric results showed that IL-37b could reduce the expression of CD203c (Figure 2f,g) but not CD63 (Figure 2e,h) induced by TSLP on basophils. The results suggested that IL-37 can reduce the TSLP-induced activation of basophils (Figure 2).

### 2.2. Depletion of Basophils Reduces the Th2 Immune Response

**Figure 3:**
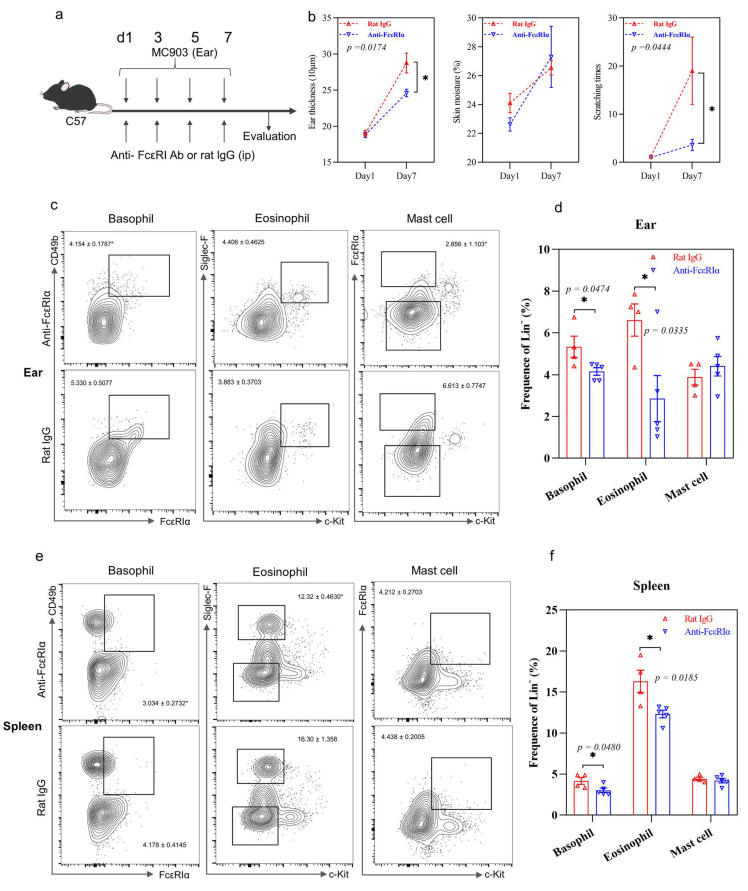

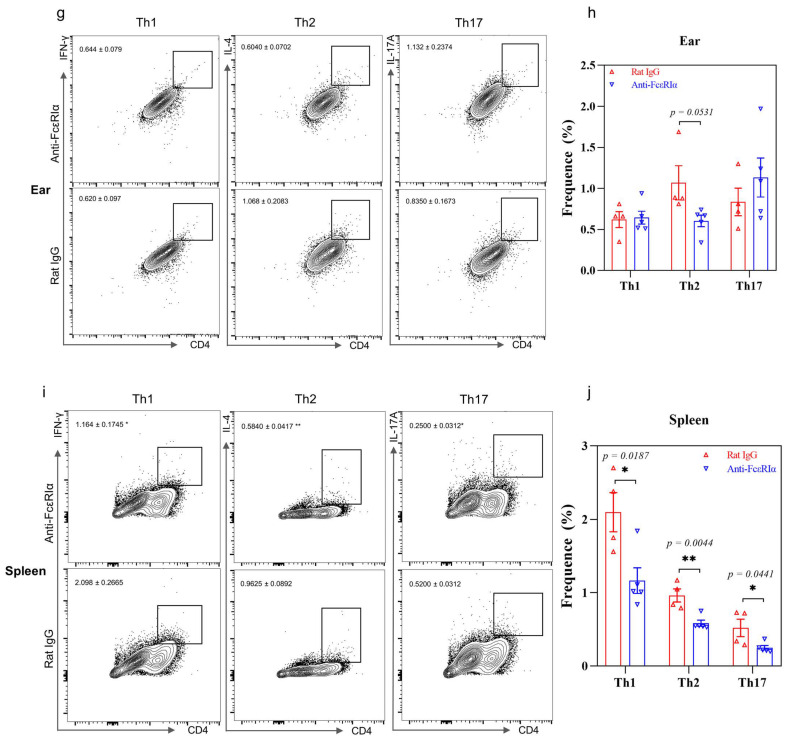
Effects of the depletion of basophils on AD. (**a**) AD murine model schedule; (**b**) ear swelling, skin moisture and scratching times; (**c**) flow cytometry analysis of basophil, eosinophil and mast cell population in the ear tissue; (**d**) quantitative analysis of result in (**c**); (**e**) flow cytometry analysis of basophil, eosinophil and mast cell population in the spleen; (**f**) quantitative analysis of results in (**e**); (**g**) flow cytometry analysis of Th1, Th2 and Th17 cell population in ear tissue; (**h**) quantitative analysis of result in (**g**); (**i**) flow cytometry analysis of Th1, Th2 and Th17 cell population in the spleen; (**j**) quantitative analysis of result in (**i**). Bar charts are shown as mean ± SEM of *n* = 4 or 5. * *p* < 0.05 and ** *p* < 0.01 when compared between the groups.

Anti-FcεRIα antibody was used to deplete basophils but not mast cells in our murine AD model (Figure 3a). The depletion of basophils significantly reduced the ear swelling and scratching times compared to the IgG antibody control treatment (Figure 3b), suggesting that basophils played an important pathogenic role in AD. The populations of basophils were also reduced in the ear and spleen, but not in lymph nodes (Figure 3c,d, Figure S2). Apart from the reduced basophils, the eosinophil populations in the ear and spleen were also significantly decreased (Figure 3c–f), suggesting a vital role of basophils in the recruitment of eosinophils. No significant changes in the basophil, eosinophil and mast cell populations in lymph nodes were observed (all *p* > 0.05, Figure S2). Importantly, the depletion of basophils resulted in a significant decrease in the Th2 cell population in the ear and spleen (Figure 3g–j) as well as Th1 and Th17 cells in the spleen, but not in lymph nodes (Figure S2), suggesting the pathogenic role of basophils in our AD murine model, for driving the naïve T cell polarization into Th2 cells.

### 2.3. TSLP-Primed Basophils in AD 

**Figure 4:**
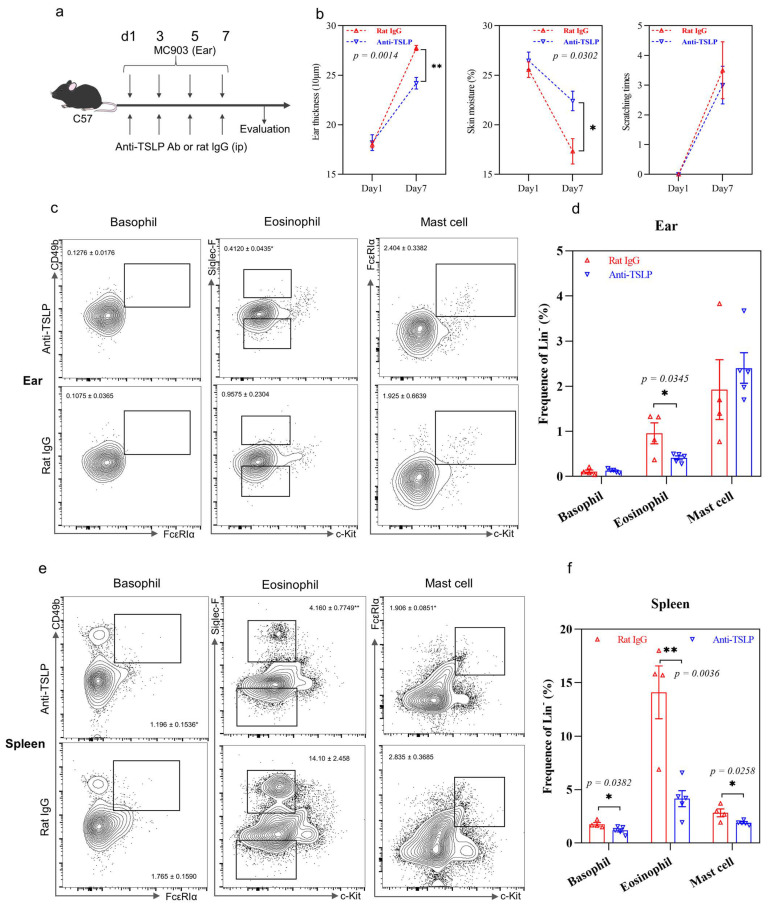

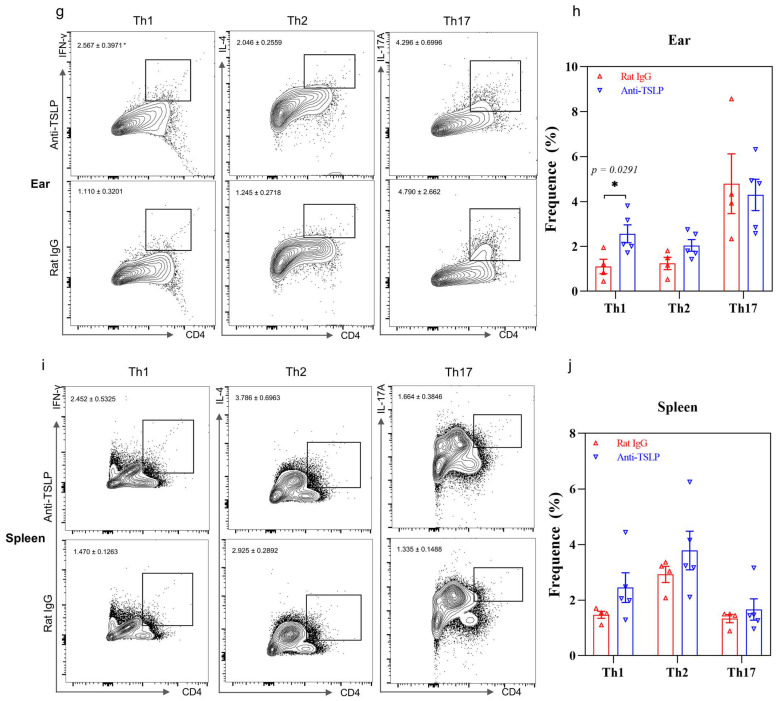
Effects of the anti-TSLP treatment on AD. (**a**) AD murine model schedule; (**b**) ear swelling, skin moisture and scratching times; (**c**) flow cytometric analysis of basophil, eosinophil and mast cell population in the ear tissue; (**d**) quantitative analysis of result in (**c**); (**e**) flow cytometry analysis of basophil, eosinophil and mast cell population in the spleen; (**f**) quantitative analysis of result in (**e**); (**g**) flow cytometry analysis of Th1, Th2 and Th17 cell population in ear tissue; (**h**) quantitative analysis of result in (**g**); (**i**) flow cytometry analysis of Th1, Th2 and Th17 cell population in the spleen; (**j**) quantitative analysis of result in (**i**). Bar charts are shown as mean ± SEM of *n* = 4 or 5. * *p* < 0.05 and ** *p* < 0.01 when compared between the groups.

An anti-TSLP antibody was used to block TSLP and to investigate the basophil population in vivo (Figure 4a). Anti-TSLP treatment significantly reduced the ear swelling and prevented skin dryness compared to IgG antibody control treatment (Figure 4b). However, no significant difference in scratching times was observed (*p* > 0.05). However, a significant decrease in basophils was observed in the spleen but not in ear tissue or lymph nodes (Figure 4c–f, Figure S3). Eosinophils and mast cells in the spleen also decreased upon anti-TSLP treatment compared to the control group (Figure 4e,f), while a significant decrease in eosinophils (Figure 4c,d) and an increase in the Th1 population (Figure 4g,h) were observed in ear tissue. No significant difference in innate cell populations in the lymph nodes was observed (*p* > 0.05, Figure S3). In line with this, no significant difference in Th2 and Th17 cell populations was observed in the ear, spleen or lymph nodes (all *p* > 0.05, Figure 4g–j and Figure S3), thereby suggesting a close link between TSLP and basophils or eosinophils but not T helper subsets. In summary, TSLP might play an important role in AD by regulating basophils and eosinophils but not directly T helper cells in vivo.

### 2.4. IL-37b Decreased the TSLP-Induced IL-4 Release from Basophils

**Figure 5:**
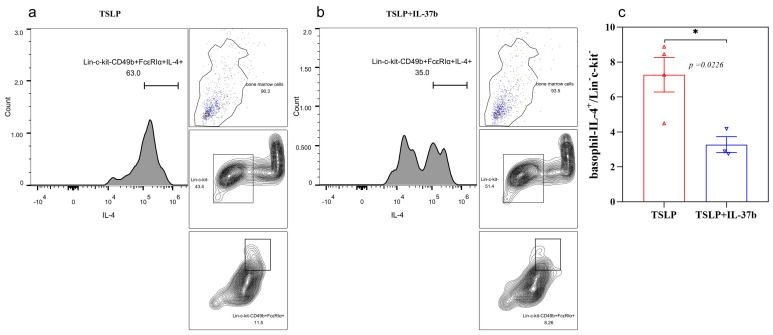
Effects of IL-37b on the IL-4 release from basophils induced by TSLP. (**a**,**b**) Flow cytometric analysis of IL-4-positive basophils in bone marrow cells; (**c**) quantitative analysis of IL-4-positive basophils in bone marrow cells. Bar charts are shown as mean ± SEM of *n* = 3 or 4. * *p* < 0.05 when compared between the groups.

TSLP can prime and activate basophils, while activated basophils can release IL-4 to initiate the Th2 immune response. In our study, basophils were induced from bone marrow cells by stimulation with IL-3 or TSLP for seven days. Cells were subsequently rested for two days, followed by TSLP restimulation overnight. The intracellular IL-4 was determined by flow cytometry. IL-37b treatment could significantly reduce the IL-4 release from TSLP-primed basophils (Figure 5a–c). Together with the results that IL-37b could directly reduce TSLP (Figure S1), we speculate that IL-37b can reduce basophil-driven IL-4 by downregulating TSLP expression.

### 2.5. IL-37b Exhibited a Protective Effect in AD by Targeting Basophils

Mice stimulated with MC903 for nine days were used to investigate the role of intrinsic immune cells in the early stages of disease development and explore the role of IL-37 in these cells (Figure S4a). If not specifically mentioned, IL-37 heterozygous transgenic mice IL-37b+/− were used. The mice applied elsewhere were IL-37 homozygous transgenic mice. In comparison to wild-type mice, IL-37b+/− exhibited less ear swelling upon stimulation, although there were no significant differences (*p* > 0.05, Figure S4b). No significant differences were observed in skin moisture and scratching in the transgenic or wild-type mice (*p* > 0.05, Figure S4b). The transgenic mice showed a significant decrease in the eosinophil population in ear tissue and lymph nodes but not in the spleen (Figure S4). IL-37b+/− also presented a decrease in Th1 and Th2 cells in ear tissue but no such difference was found in the spleen or lymph nodes (Figure S4). Generally, basophils would respond earlier than eosinophils in allergic diseases. However, no significant differences in basophil populations were observed in the ear, spleen or lymph nodes, suggesting that the endpoint of the current model is belated. Together, IL-37b+/− can exhibit protective effects on the development of AD-mediated allergic inflammation. 

**Figure 6:**
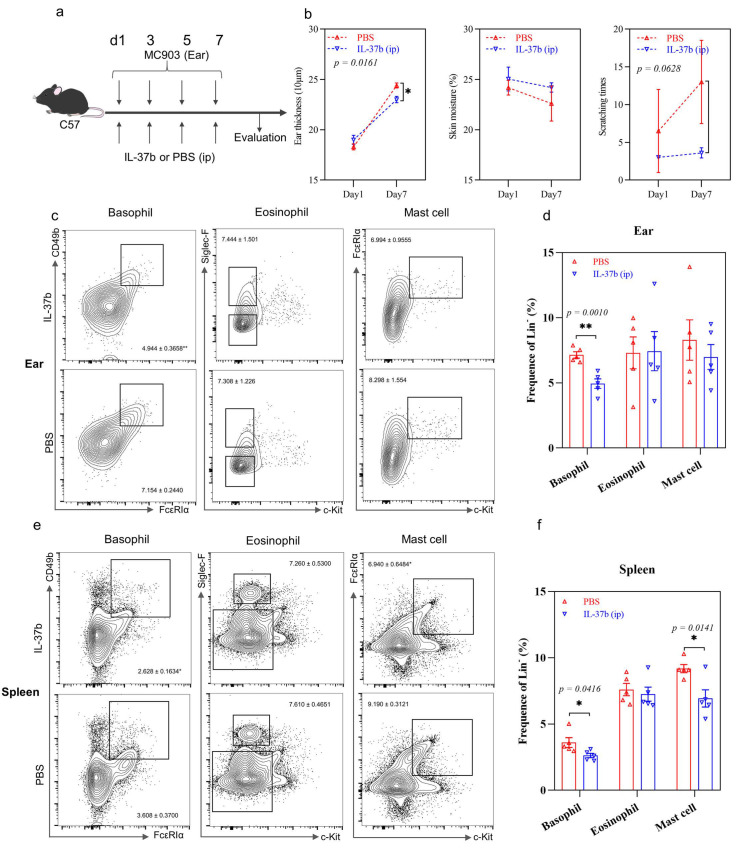

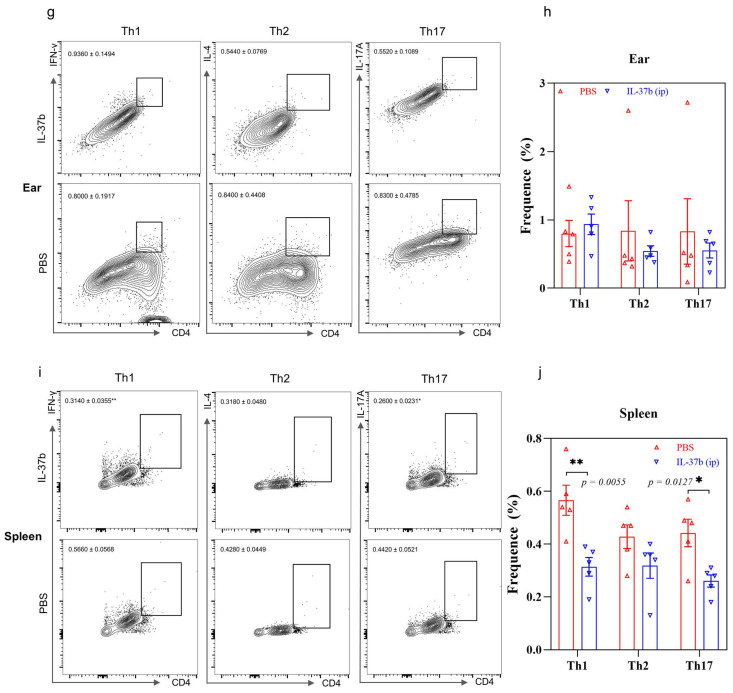
Effects of IL-37b injection on AD. (**a**) AD murine model schedule; (**b**) ear swelling, skin moisture and scratching times; (**c**) flow cytometry analysis of basophil, eosinophil and mast cell population in ear tissue; (**d**) quantitative analysis of result in (**c**); (**e**) flow cytometry analysis of basophil, eosinophil and mast cell population in the spleen; (**f**) quantitative analysis of result in (**e**); (**g**) flow cytometry analysis Th1, Th2 and Th17 cell population in ear tissue; (**h**) quantitative analysis of result in (**g**); (**i**) flow cytometry analysis Th1, Th2 and Th17 cell population in the spleen; (**j**) quantitative analysis of result in (**i**). Bar charts are shown as mean ± SEM of *n* = 4 or 5. * *p* < 0.05 and ** *p* < 0.01 when compared between the groups.

We next directly treated mice with human recombinant IL-37b 1 h prior to MC903 stimulation to investigate the role of IL-37 in the innate immune cells in our murine AD model, with a 7-day modified model (Figure 6a). Mice with IL-37b treatment showed a significant decrease in ear swelling compared to PBS treatment (Figure 6b), but no significant differences in skin moisture or scratching times were observed. Of note, IL-37b treatment significantly reduced the basophil population in the ear and spleen (Figure 6c–f). IL-37 treatment also significantly reduced the mast cell population in the spleen (Figure 6e,f). The decreased basophils and mast cell population might account for the reduced scratching time (*p* = 0.0628) in the IL-37b treatment group compared with PBS treatment (Figure 6b–f), which was consistent with the result upon anti-FcεRIα treatment (Figure 3b). No significant difference in the cell population in lymph nodes was observed (*p* > 0.05, Figure S5). Along with the decreased basophil population, IL-37b-treated mice showed a significant decrease in Th1 and Th17 cell populations in the spleen (*p* < 0.05) and a decrease in the Th2 population in the ear and spleen (*p* > 0.05) (Figure 6g–j). There were no significant differences in Th1, Th2 or Th17 cells in the lymph nodes (all *p* > 0.05, Figure S5). In summary, IL-37b treatment showed a protective role in AD-mediated allergic inflammation by reducing basophils.

**Figure 7:**
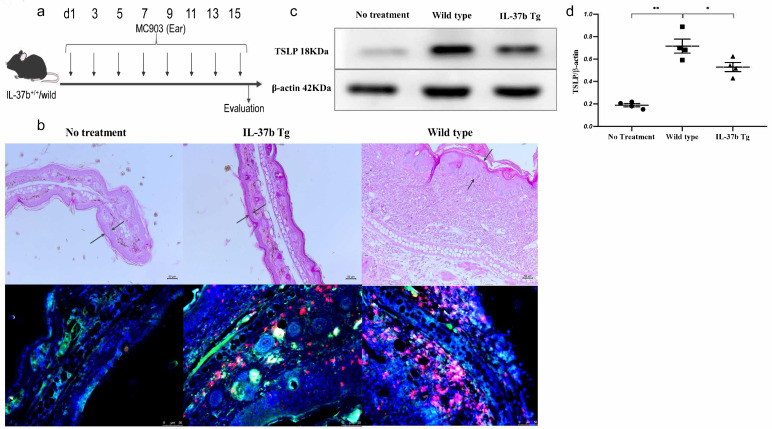
Effects of the IL-37b Tg (homogeneous) on AD. (**a**) AD murine model schedule; (**b**) H&E staining and immunofluorescence staining of MBP (red) and TSLP (green) of ear tissue; (**c**) Western blot analysis of TSLP in ear tissue; (**d**) quantitative analysis of TSLP expression. Bar charts are shown as mean ± SEM of *n* = 4 or 5. * *p* < 0.05 and ** *p* < 0.01 when compared between the denoted groups.

Moreover, we adopted IL-37b homogeneous transgenic mice to investigate the TSLP expression and basophil infiltration in the ear skin tissue (Figure 7a). The IL-37b Tg mice showed a reduction in ear swelling (Figure 7b). Of note, the immunofluorescence staining results demonstrated that IL-37b Tg could reduce the expression of TSLP (green) and basophil (red) infiltration in ear tissue (Figure 7b). Importantly, the significant decrease in TSLP expression in the ears of IL-37b Tg mice was verified by using Western blot, by comparison with wild-type mice (Figure 7c,d and Figure S6). Together with TSLP’s priming activity on basophil activation and the in vitro suppressive activity of IL-37b on TSLP expression, we conclude that IL-37b could ameliorate the allergic inflammation in AD by regulating TSLP-activated basophils.

## 3. Discussion

AD is a pruritic inflammatory and chronic relapsing skin disease. AD patients present with the accumulation and infiltration of antigen-specific IgE-activated basophils [15,16]. Recent studies have shown basophil accumulation in skin lesions of various skin diseases in humans, including AD, urticaria, pruritus and bullous pemphigoid, Stevens–Johnson syndrome, eosinophilic pustular folliculitis and IgA vasculitis [22].

Basophils have attracted attention regarding their central role in the pathogenesis of allergic diseases [8,16,23]. Basophils are terminally differentiated granulocytes without proliferation. This maturation process critically depends on the expression of transcription factors C/EBPα and GATA-2 [24] and the action of IL-3 and/or TSLP [8,9]. Basophils express high-affinity IgE receptors (FcεRI) and rapidly release variously performed histamine, basogranulin, vascular endothelial growth factor (VEGF) and de novo synthesized mediators (Leukotriene C4, chemokines/cytokines such as IL-4 and IL-13) in response to IgE cross-linking (antigens, superantigens, anti-IgE) or independence of IgE stimulation (cytokines, anti-IgD, anaphylatoxins, proteases, TLR ligands) [25,26].

The integrity of the skin is essential for the maintenance of normal skin function. However, when the skin is stimulated by antigens, keratinocytes produce inflammatory mediators such as TSLP, which will recruit and activate basophils. The activated basophils will release Th2 cytokine IL-4, which then induces naïve T cell differentiation into Th2 cells for the Th2 immune response. The alternative depletion of basophils ameliorated the AD symptoms with a decreased Th2 population, indicating the potential role of basophils in the initiation of the Th2 immune response (Figure 3b,g–j). In this study, the ex vivo activation of basophils by TSLP showed that basophils could release IL-4, a key mediator of Th2 immune response initiation (Figure 5). These results indicate that basophils were partly responsible for the Th2 immunity. Since basophils can account for inducing pruritis in AD [27], the alternative depletion of basophils showed a significant decrease in the scratching behavior and basophil population, but not mast cells, compared to the control mice (Figure 3b–d). This indicated that basophils played a role in the pathogenesis of AD, especially in triggering itchiness. The eosinophil populations in the ear and spleen were also reduced by the depletion of basophils, thereby suggesting the effect of basophils on the recruitment of eosinophils to the challenged tissue (Figure 3c–f). 

However, blockage of TSLP only significantly reduced the basophil population in the spleen but not in the ear (Figure 4c–f). Since basophils can be activated either by TSLP or IL-3, blocking the function of basophils by TSLP alone may not be sufficient. Blockage of TSLP ameliorated the skin dryness but not scratching behavior (Figure 4b), which is consistent with the unchanged basophil population in the ear tissue (Figure 4c). The correlation of basophil number with scratching times partly demonstrated that basophils might cause itchiness, as observed in AD patients.

Our previous study has shown that IL-37b transgenic mice express the IL-37b protein before and after the induction of AD, whereas wild-type mice do not [28]. We found that IL-37b could significantly reduce TSLP expression to inhibit the Th2 immune response both in vitro and in vivo. Firstly, IL-37b could reduce the expression of the TSLP receptor on basophils (Figure 1a,b), subsequently reducing the activation of basophils. IL-37b reduced the IL-4 from mouse basophils upon TSLP stimulation ex vivo (Figure 5). Secondly, although IL-37b+/− mice showed no significant difference in basophil population or AD symptoms, a significant decrease in the eosinophil population in the ear and lymph nodes and the Th2 cell population in the ear were observed (Figure S4). Meanwhile, IL-37b+/− mice showed no difference in scratching behavior compared to wild-type mice, probably due to the unchanged basophil population (Figure S4c,d). The poor anti-inflammatory effects observed in IL-37b+/− mice may be due to insufficient expression of IL-37b in the IL-37 heterozygous transgenic mice. In our previous study, AD patients showed a significant decrease in serum levels of IL-37 compared to healthy controls [29], in compliance with the significant reduction in IL-37 expression in skin lesions of AD patients, as revealed by other groups [30,31]. In parallel, IL-37 has been reported to be reduced in allergic or inflammatory diseases [32,33,34]. Immune flare-ups following allergen stimulation may result from inadequate immunosuppression by IL-37, an essential part of AD pathology. Therefore, optimal production and activity of IL-37 are essential to maintain the balance of the immune response.

Finally, IL-37b intraperitoneal (ip) treatment reduced the basophil infiltration in the ear and spleen and the Th2 immune response. IL-37b (ip) also significantly reduced the scratching times in AD mice compared to control mice (Figure 6). Therefore, IL-37b could ameliorate AD itching status by targeting basophils. Due to the difference in immunological features between IL-37b knock-in mice and IL-37b-injected mice, we should carefully interpret the data and draw conclusions with much caution. Our previous study [28] also observed that IL-37b homogeneous transgene mice presented with a significant decrease in the scratching time on day 7. Both in vitro and ex vivo experiments showed that IL-37b has a regulatory role in basophils by modulating TSLP expression. Furthermore, IL-37b homogeneous transgenic mice showed a decrease in TSLP expression along with reduced infiltration of basophils, confirming that IL-37 could ameliorate AD by regulating TSLP-mediated basophil activation (Figure 7). Either treatment of IL-37b by intraperitoneal or homogeneous transgene presented an improvement in AD symptoms such as ear swelling and itching status. In conclusion, IL-37b could play a crucial role in regulating TSLP-primed basophils, thereby suggesting that IL-37b could be a promising agent for the treatment of AD.

## 4. Materials and Methods

### 4.1. Reagents

Recombinant human TSLP was purchased from PeproTech, Rocky Hill, NJ, USA. Recombinant Der P1 protein was purchased from ProSpecTany, Rehovot, Israel. Human IL-37b was obtained from R&D system, Minneapolis, MN, USA. APC anti-human CD63 (H5C6), FITC-anti-human CD203c (E-NPP3), PE anti-human Phospho-STAT6 (A15137E), PE-anti-human TSLPR (1B4) and APC anti-human CD127 (A019D5) were purchased from BioLegend, San Diego, CA, USA. 

Purified anti-mouse FcεRIα, purified anti-mouse TSLP and their related rat IgG antibodies were purchased from BioLegend. Mouse regulatory T cell staining kit was obtained from eBioscience, San Diego, CA, USA, which included fluorescein isothiocyanate (FITC)-conjugated rat anti-mouse CD4 monoclonal antibody, APC-conjugated rat anti-mouse CD25 monoclonal antibody, PE-conjugated rat anti-mouse Foxp3 monoclonal antibody, PE-conjugated rat IgG2a isotypic control, anti-mouse CD16/32 (Fc block), flow cytometry staining buffer, fixation/permeabilization concentrate and diluent, as well as permeabilization buffer (10×). FITC anti-mouse CD3 (17A2), CD4 (GK1.5), CD5 (53–7.3), CD8 (53–6.7), CD19 (1D3); PE/Cyanine7 anti-mouse CD49b (HMα2), IL-17A (TC11-18H10.1); Alexa Fluor^®^ 700 anti-mouse FcεRIα (MAR-1); APC anti-mouse Siglec-F (S17007L), CD63 (H5C6), IL-4 (11B11); PE anti-mouse c-Kit (2B8), IFN-γ (XMG1.2) were purchased from BioLegend. In addition, 7-AAD viability staining solutions were purchased from BioLegend. 

Human HaCaT cells, spontaneously transformed keratinocytes from histologically normal human skin in vitro, were purchased from CLS Cell Lines Service GmbH, Eppelheim, Germany. HaCaT cells were cultured in DMEM medium (Thermo Fisher Scientific Inc., Rockford, IL, USA) supplemented with 4.5 g/L glucose, 2 mM L-glutamine and 10% heat-inactivated fetal bovine serum (FBS, Gibco, Waltham, MA, USA). KU812 basophilic cells isolated from chronic myelogenous leukemia were obtained from ATCC (Manassas, VA, USA) and maintained in RPMI1640 (Thermo Fisher) supplemented with 10% FBS.

MC903 (calcipotriol, also known as calcipotriene, is a synthetic derivative of calcitriol, a form of vitamin D, low-calcemic vitamin D3 analog), a TSLP inducer, was from Bio-techne, Minneapolis, MN, USA and was dissolved in ethanol to make the stock concentration of 20 mM and kept at −80 °C in the dark. The working solution was prepared by diluting the concentrate with ethanol to 0.2 nmol/μL. 

### 4.2. Purification of Basophils

Fresh human buffy coat donated by healthy volunteers was obtained from the Hong Kong Red Cross Blood Transfusion Service. After 1:2 dilution with PBS, a fresh human buffy coat was added to the top of the 1.082 g/mL isotonic Ficoll solution and centrifuged for 25 min at 1800 rpm, room temperature. PBMC fractions were washed two times and then incubated at 4 °C for 10 min with FcR blocking reagent and Basophil Biotin-Antibody Cocktail (MACS, Miltenyi Biotec, Bergisch Gladbach, Germany). The mixture was then incubated with anti-biotin microbeads for an additional 15 min at 4 °C (Miltenyi Biotec). The basophil-enriched drop-through fraction was collected via the depletion of non-basophils by loading the cells onto an LS column within a magnetic field (Miltenyi Biotec.). The purity of basophils ranged from 85% to 95%, as assessed by Hemacolor rapid blood smear staining. Following the 1964 Declaration of Helsinki and its subsequent revisions, the experimental procedures using human materials were approved by the Clinical Research Ethics Committee of the joint Chinese University of Hong Kong–New Territories East Cluster. (CREC Ref. No.: 2017.614, 1 January 2018).

### 4.3. Co-Culture 

Human keratinocyte cell line HaCaT cells were maintained in DMEM medium supplemented with 10% FBS. KU812 (3 × 10^5^ cells) and confluent HaCaT (1 × 10^5^ cells) cells were co-cultured in RPMI 1640 medium containing 10% FBS or incubated alone with or without pretreatment with IL-37b (100 or 200 ng/mL) for 10 min and then stimulated with Der p1 (1 μg/mL, a cysteine proteinase derived from the allergen house dust mite) for 24 h before protein expression was determined.

### 4.4. Animals and AD Murine Model

Inbred CRISPR/Cas9 human IL-37b knock-in mice (8 weeks old; 57BL/6 background) were obtained from Cyagen Biosciences (Guangzhou, China). IL-37b knock-in mice and C57 mice were bred under SPF conditions and housed at the Chinese University of Hong Kong Laboratory Animal Service Center. All animal experiments were performed in compliance with the guidelines in the Animal Experimentation Ethics Committee (AEEC) Guide for the Care and Use of Laboratory Animals approved by the AEEC of the Chinese University of Hong Kong. (Ref No.: 17-181-GRF, 15 December 2017).

MC903-induced AD-like mice model was established according to our previous publications [35]. A total of 2 nmol of MC903 dissolved in 10 μL ethanol was topically applied to the right ear skin of each mouse (1 nmol per side; 5 μL) every two days for 7, 9 or 16 days (four, five or nine times in total). The left ear was given 10 μL ethanol as self-control in the 16-day cohort. Mice in the normal control group were given ethanol to both ears. Both left and right ears were stimulated with MC903 in the 7-day cohort.

In the 7-day cohort, mice were injected with anti-TSLP (20 μg/mouse, four times; Biolegend) or rat-IgG (20 μg/mouse, four times; Biolegend), anti-FcεRIα (20 μg/mouse, four times; Biolegend) or rat-IgG (20 μg/mouse, four times; Biolegend), and IL-37b (2 μg/mouse, four times; R&D) or PBS was administrated by intraperitoneal injection 1 h prior to MC903 stimulation. Mice were terminated on day 8, 10 or 17 and EDTA anticoagulated blood was collected; then, ears, spleen and skin lymph nodes were removed for the following investigations.

Ear thickness was monitored every other day before the MC903 challenge and treatment using a dial thickness gauge (Model G, Peacock, Ozakimfg Co, Ltd., Tokyo, Japan). The change in thickness before and after ear stimulation was recorded as ear swelling. Scratching time within 3 or 5 min was documented every other day to evaluate the severity of itching for 7, 9 or 16 days, respectively. Then, mice were sacrificed for post-mortem analysis of AD skin lesions. Rapid and consecutive multiple scratches over a brief period were considered one-time scratches.

### 4.5. Preparation of Single-Cell Suspensions and Flow Cytometric Analysis

The single spleen cell and skin lymph node suspensions were prepared by grinding the spleen on a 70 μm cell strainer (BD Biosciences) connected to a 50 mL conical tube with the plunger of a 3 mL syringe and washing buffer (1 × PBS supplemented with 2 mM EDTA and 2% FBS). After RBC lysis, splenocytes and lymph node cells were washed and resuspended in PRMI1640 medium supplemented with 10% FBS.

The chopped ear tissue was digested in DMEM media for 90 min at 37 °C by 0.25 mg/mL Liberase TL (Roche) followed by mashing through 70 μm cell strainers. Samples were then washed with DMEM media supplemented with 5% FBS, 1% L-glutamine (GIBCO) and 1% penicillin/streptomycin (GIBCO). Single-cell suspensions were prepared for subsequent staining for flow cytometry. 

Bone marrow cells (5 × 10^6^ cells/mL) were obtained from WT C57BL/6 mice and maintained in Mast Cell Medium (RPMI 1640, 15% FBS, 100 U/mL penicillin, 100 μg/mL streptomycin, 2.9 mg/mL glutamine, 50 mmol/L 2-mercaptoethanol, 1 mmol/L sodium pyruvate, 1× non-essential amino acids and 10 mmol/L HEPES). TSLP-dependent or IL-3-dependent basophils were ex-vivo-induced by recombinant TSLP (1 μg/mL; PeproTech, Rocky Hills, NJ, USA) or IL-3 (10 U/mL, Biolegend) for five days, with medium replenished daily. For the analysis of the population of IL-4+, IL-17+ and IFN-γ+ cells in CD4+ cells, cells were first stained with anti-CD4 antibody for 30 min at 4 °C. After washing, cells were fixed and permeabilized using fixation/permeabilization working solution for 30 min at room temperature in the dark. Then, cells were stained with IL-4, IL-17A, IFN-γ and Foxp3 antibodies for another 30 min at room temperature in the dark after washing using 1× permeabilization buffer two times. After the last washing, cell pellets were re-suspended in flow cytometry staining buffer and analyzed by BD FACSCalibur flow cytometer (BD Biosciences, San Jose, CA, USA). 

For the analysis of the population of basophils, eosinophils and mast cells, single cells were stained with the surface markers for 30 min at room temperature. After the last washing, cell pellets were resuspended in flow cytometry staining buffer and analyzed by Navios EX Flow Cytometer (Beckman Coulter, Miami, FL, USA). Mouse basophils were identified as live, Lin- (CD3, CD4, CD5, CD8, CD19) c-kit-, Siglec-F-, FcεRIα+, CD49b+; mouse eosinophils were identified as live, Lin- (CD3, CD4, CD5, CD8, CD19), c-kit-, Siglec-F+; mast cells were identified as live, Lin- (CD3, CD4, CD5, CD8, CD19), c-Kit+, FcεRIα+ cells. The flow cytometry gating strategies are presented in Figure S7.

### 4.6. Quantitative Polymerase Chain Reaction, Histological Analysis and Western Blot

The detailed methods of quantitative polymerase chain reaction, histological analysis and Western blot are described in supplementary materials. The primer sequences used are listed in Table S1.

### 4.7. Statistical Analysis

Data were presented as mean ± SEM and analyzed by unpaired Student *t* test or a nonparametric Mann–Whitney test (when the normal distribution of the data could not be guaranteed) or one-way analysis of variance (ANOVA) followed by Dunnett’s test for multiple inter-group comparisons, using GraphPad PRISM software version 8.01. *p* < 0.05 was considered statistically significant.

## Data Availability

The data presented in this study are available on request from the corresponding author.

## Supplementary Materials

The following are available online at https://www.mdpi.com/article/10.3390/ijms22147393/s1.
