# Supplementary material for: IL-37 Targets TSLP-Primed Basophils to Alleviate Atopic Dermatitis
Source: Int J Mol Sci. 2021 Jul 9;22(14):7393. doi: 10.3390/ijms22147393 (PMC8304334; doi:10.3390/ijms22147393)
Supplement: Supplementary file 1 [file ijms-22-07393-s001.zip › ijms-1284496-supplementary.pdf]

International Journal of *Molecular Sciences*

MDPI

Supplementary Materials

IL-37 targets TSLP-primed basophils to alleviate atopic dermatitis

Tianheng Hou[1], Miranda Sin-Man Tsang[1,2], Lea Ling-Yu Kan[2], Peiting Li[2], Ida Miu-Ting Chu[1], Christopher Wai-Kei Lam[3] and Chun-Kwok Wong[1,2*]

[1] Department of Chemical Pathology, The Chinese University of Hong Kong, Prince of Wales Hospital, Hong Kong, China
[2] Institute of Chinese Medicine and State Key Laboratory of Research on Bioactivities and Clinical Applications of Medicinal Plants, The Chinese University of Hong Kong, Hong Kong, China
[3] Faculty of Medicine and State Key Laboratory of Quality Research in Chinese Medicines, Macau University of Science and Technology, Macau, China

Methods

1. Quantitative polymerase chain reaction

Total RNA of HaCaT cells (3 × 105 cells/well; 24 well, alone cultured) and basophils (4.2 × 105 cells/well; 24 well, cultured alone) isolated from human buffer coat was extracted using an RNA extraction kit (Qiagen Corp., Germantown, MD, United States). The relative expressions of IL-4, IL-37b, IL-18Rα, IL-1R8, Smad3, TSLP and TNF-α were determined by using 2-ΔCt (Ct, target gene-Ct, GAPDH). Primer sequences used were listed in table S1.

2. Histological analysis

Ear tissues were obtained at the end of the experiment, then fixed with 10% neutral buffered formalin, dehydrated and embedded in paraffin. Sections (5 μm) were prepared for the following staining. After deparaffinization and hydration, ear sections were stained with hematoxylin and eosin, followed by dehydration and mounted with Canada balsam mounting medium (Sigma-Aldrich). For assessing the general morphology, ear sections were examined under a Leica DM6000 B microscope (Leica Microsystems Inc.) and processed by Leica Application Suite software.

Deparaffinized ear sections were subjected to antigen retrieval by immersing slides in sodium citric buffer (0.01 M, pH 6.0) and heating to 100°C for 5 min in a microwave oven. Then the slides were cooled at room temperature for 30 min. After blocking with 5% FBS for 30 min at room temperature, slides were incubated with the first antibodies with the dilution at 1:200 in 1×TBS supplemented with 1% BSA overnight at 4°C. After washing with 1×TBST (TBS supplemented with 0.1% tween-20), slides were incubated with a secondary antibody with the dilution at 1:500 in 1×TBS supplemented with 1% BSA for 1 h at room temperature. After washing with TBST, slides were counterstained with DAPI (1:1000) for 5 min at room temperature prior to microscopic examination. After washing and mounting, slides were read under the Leica DM6000 B microscope (Leica Microsystems GmbH, Wetzlar, Germany). Rat anti-mouse mast cell protease (MCP)-8 antibody specific for basophils were from Biolegend. TSLP rabbit polyclonal antibody were obtained from ABclonal Science, Inc Woburn, MA, USA. Cy3-conjugated goat anti-rat immunoglobulin G (IgG) antibody was purchased from Beyotime Co., Shanghai, China. Alexa Fluor 488-conjugated goat anti-rabbit IgG antibody were purchased from ABclonal, used as secondary antibodies. 4',6-diamidino-2-phenylindole (DAPI) for nucleus staining was purchased from Invitrogen, Carlsbad, USA.

3. Western blot

Cells from co-cultures or homogenized mouse ear were lysed with RIPA lysis buffer (Thermo Fisher Scientific) containing protease inhibitor and phosphatase inhibitor (Sigma-Aldrich). Protein concentration was determined by Pierce BCA protein assay (Thermo Fisher Scientific). Samples (20 μg) were loaded into each well and separated on 10% or 12% SDS-polyacrylamide gels. Targeted proteins were transferred to polyvinylidene fluoride membranes (Immobilon-PSQ; Millipore, MA, USA) and PVDF membranes were blocked with 5% bovine serum albumin (BSA) in Tris-buffer saline/0.1% Tween 20 (TBST) for 1 hr at room temperature. The membrane was then incubated with primary TSLP antibodies (ABclonal Science) diluted 1:500 or 1:1000 in 5% BSA-TBST overnight at 4°C. After washing with TBST, the membrane was incubated with peroxidase (HRP)-conjugated anti-rabbit secondary antibody (Biolegend) diluted in TBST with 5% BSA for 1 h at room temperature. After the last washing, the immune complexes were visualized using the enhanced chemiluminescence (ECL) method (GE Healthcare, Piscataway, NJ, USA).

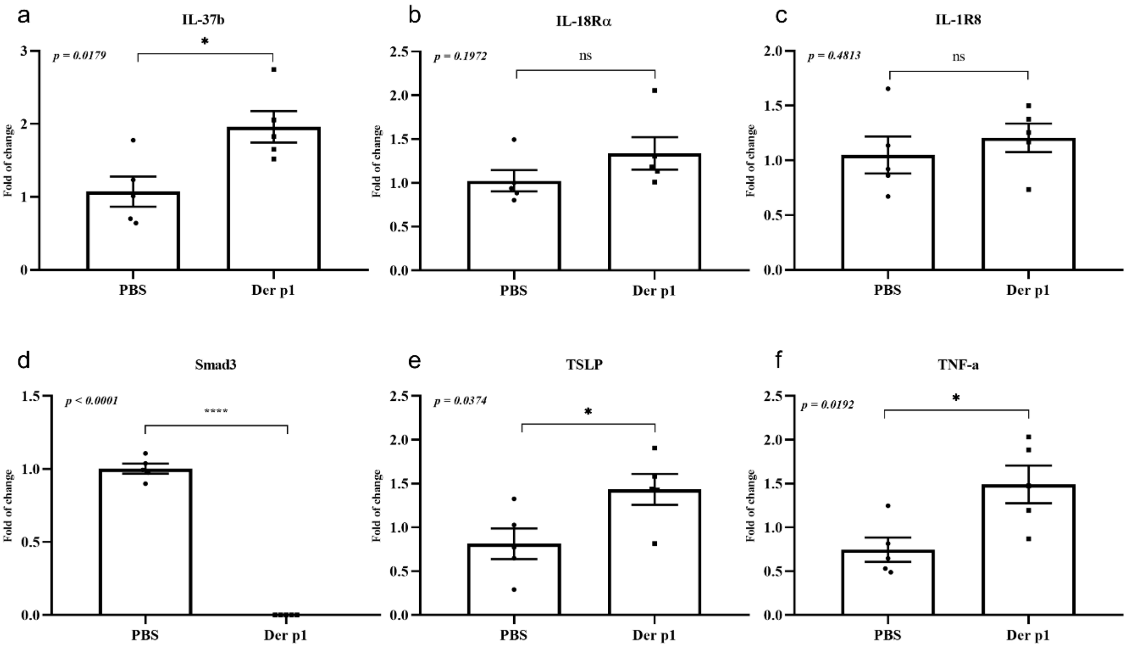

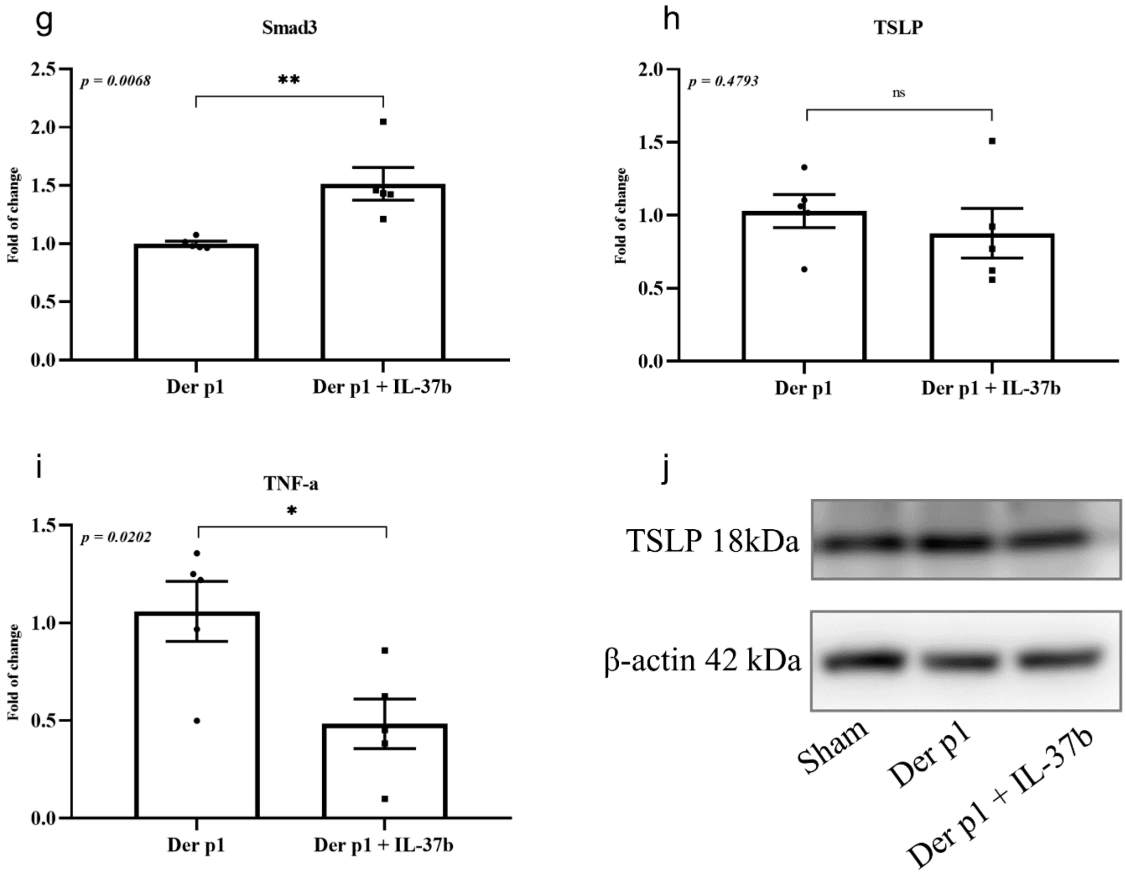

Figure 1. *In vitro* effects of IL-37b on the inflammation induced by Der p1 in HaCaT cells. Der p1 induced the dysregulation of (**a**) IL-37b, (**b**) IL-18Rα, (**c**) IL-1R8, (**d**) Smad3, (**e**) TSLP, and (**f**) TNF-α; IL-37b reversed the dysregulation of (**g**) Smad3, (**h**) TSLP and (**i**) TNF-α; (**j**) Western blots analysis of TSLP expression upon different treatment. Bar charts are shown as mean ± SEM of n=5. *P < 0.05 and **P < 0.01 when compared between the denoted groups.

a

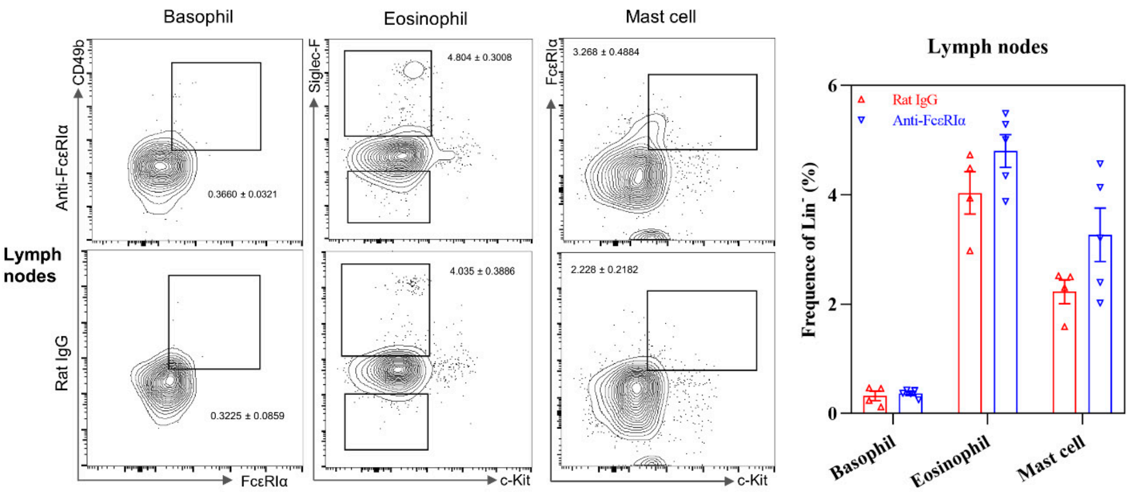

b

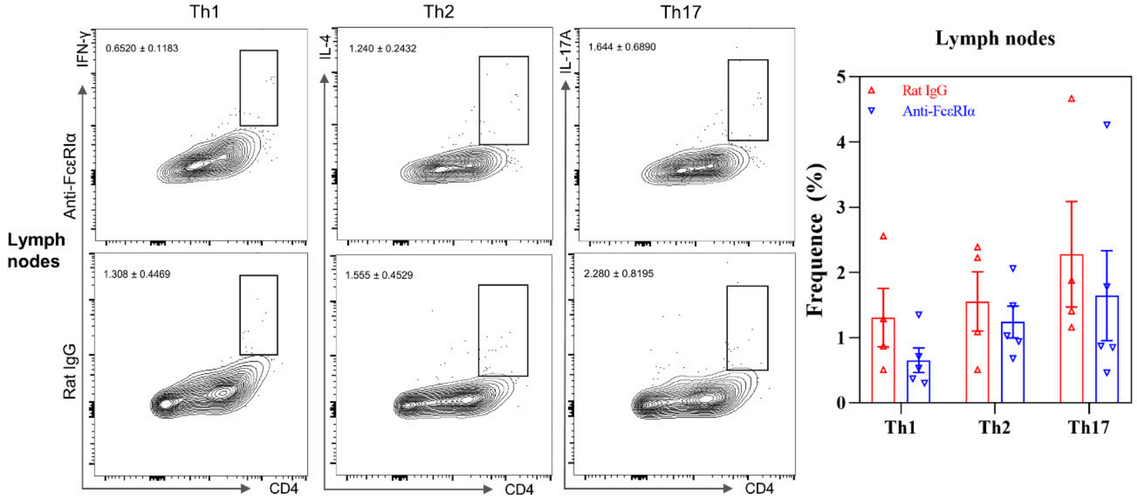

Figure 2. *In vivo* effects of the depletion of basophils on AD. (**a**) Flow cytometry analysis and quantitative analysis of basophil, eosinophil and mast cell population in the lymph nodes; (**b**) Flow cytometry analysis and quantitative analysis of Th1, Th2, and Th17 cell population in the lymph nodes. Bar charts are shown as mean ± SEM of n= 4 or 5.

a

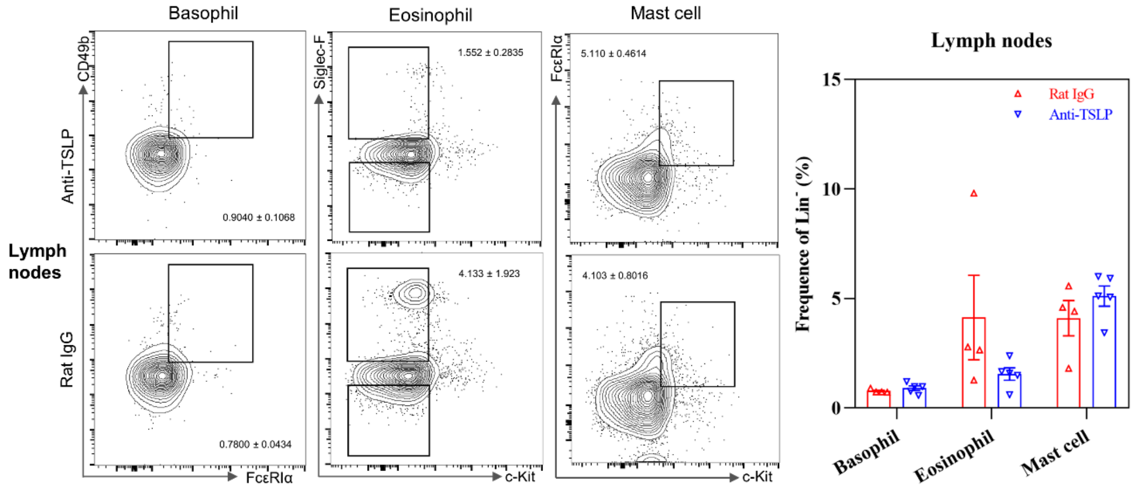

b

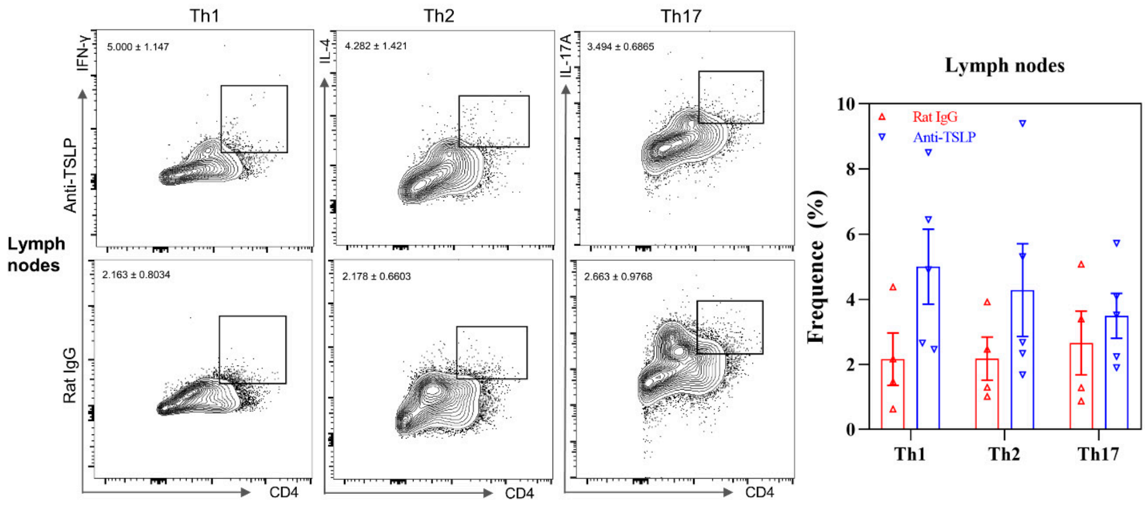

Figure 3. *In vivo* effects of the treatment of anti-TSLP on AD. (**a**) Flow cytometry analysis and quantitative analysis of basophil, eosinophil and mast cell population in the lymph nodes; (**b**) Flow cytometry analysis and quantitative analysis of Th1, Th2 and Th17 cell population in the lymph nodes. Bar charts are shown as mean ± SEM of n= 4 or 5.

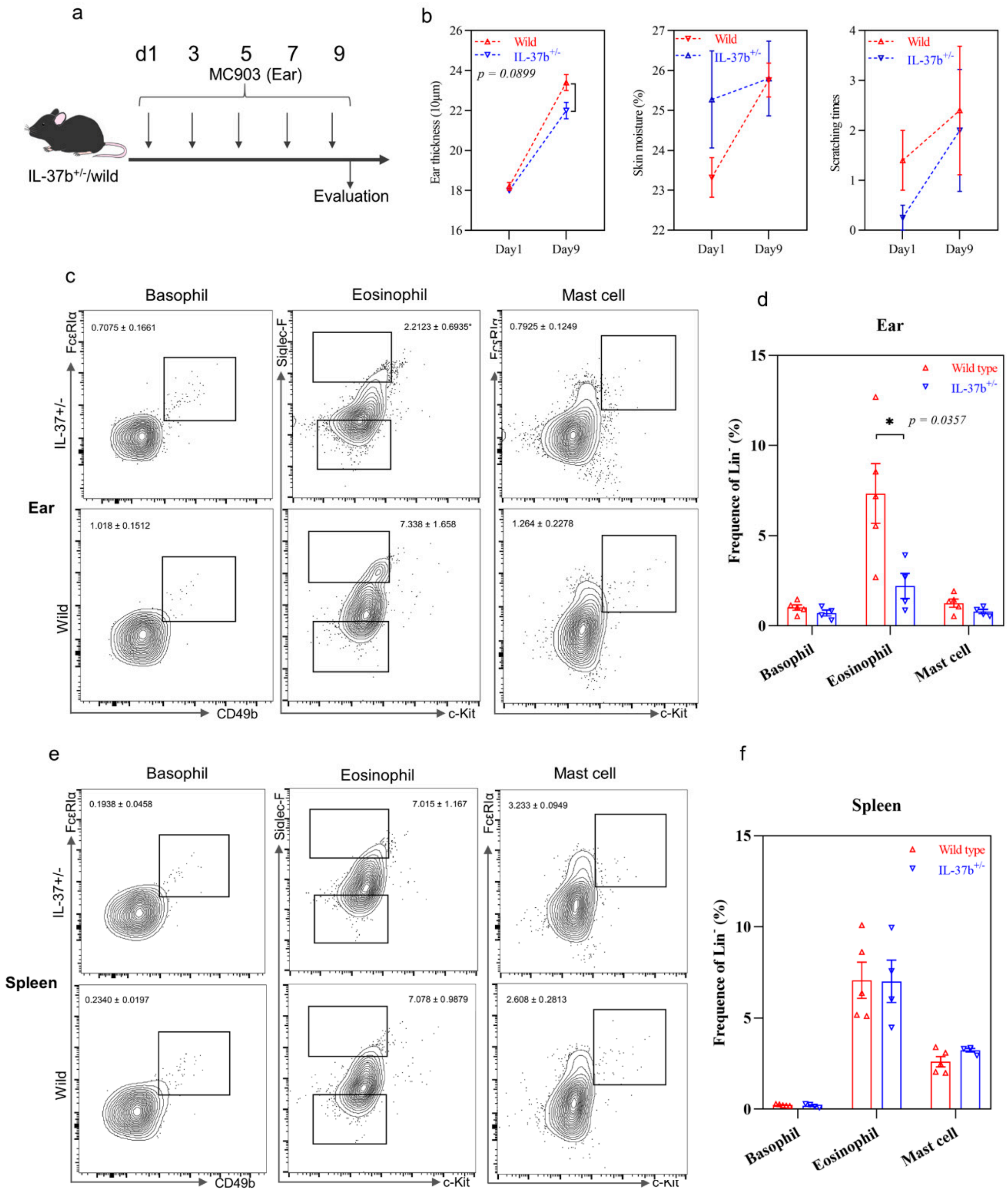
a
d1 3 5 7 9
MC903 (Ear)
IL-37b+/-/wild
Evaluation
b
Wild
IL-37b+/-
p = 0.0899
Ear thickness (10μm)
Skin moisture (%)
Scratching times
Day1 Day9
c
Basophil
Eosinophil
Mast cell
Ear
IL-37+/-
Wild
0.7075 ± 0.1661
2.2123 ± 0.6935*
0.7925 ± 0.1249
1.018 ± 0.1512
7.338 ± 1.658
1.264 ± 0.2278
FcεRIα
Siglec-F
CD49b
c-Kit
d
Ear
Wild type
IL-37b+/-
p = 0.0357
Frequence of Lin- (%)
Basophil
Eosinophil
Mast cell
e
Spleen
0.1938 ± 0.0458
7.015 ± 1.167
3.233 ± 0.0949
0.2340 ± 0.0197
7.078 ± 0.9879
2.608 ± 0.2813
f
Spleen

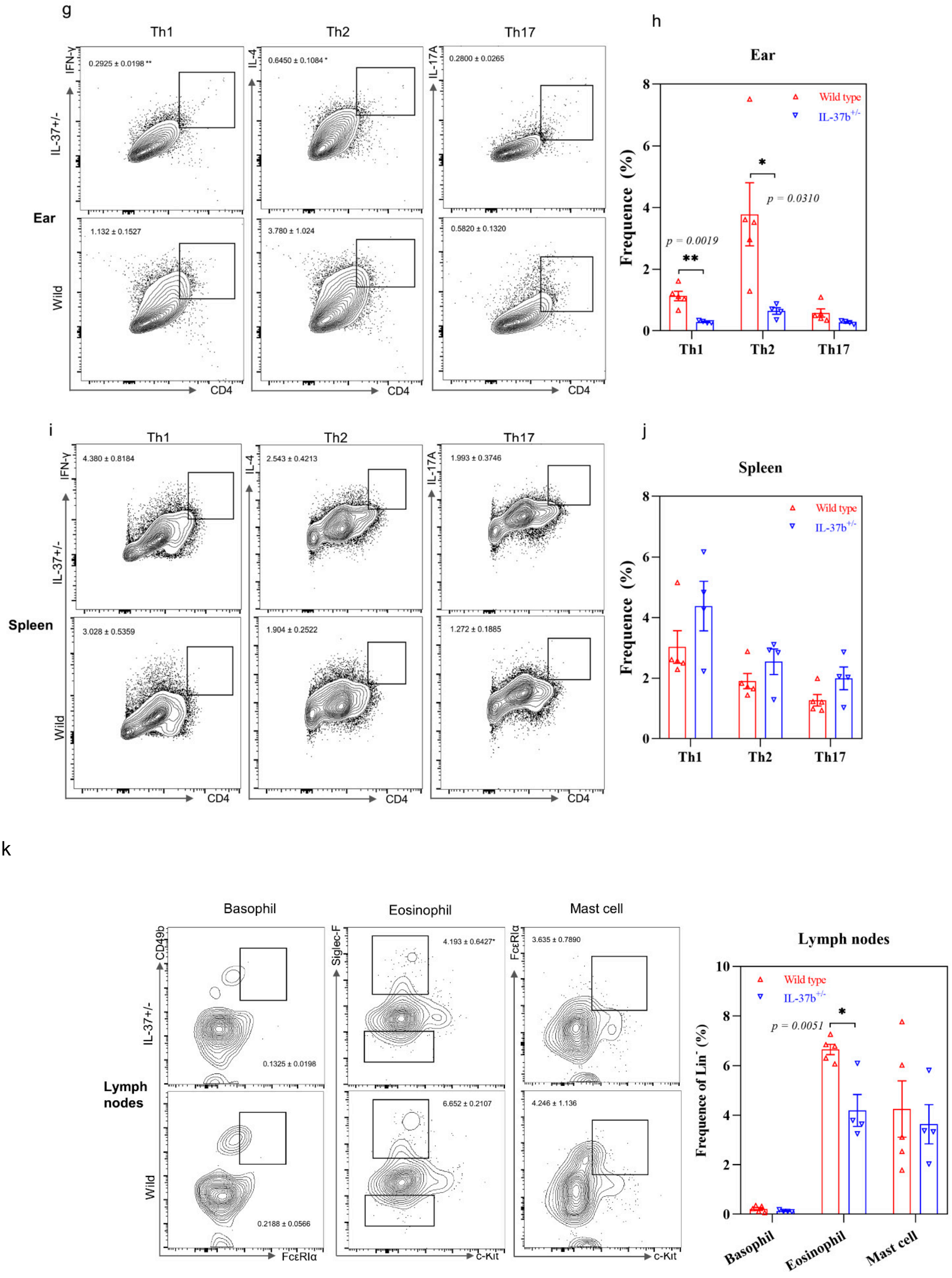
g
Th1
Th2
Th17
IFN-γ
IL-4
IL-17A
0.2925 ± 0.0198 **
0.6450 ± 0.1084 *
0.2800 ± 0.0265
IL-37+/-
Ear
1.132 ± 0.1527
3.780 ± 1.024
0.5820 ± 0.1320
Wild
CD4
h
Ear
Wild type
IL-37b+/-
Frequence (%)
p = 0.0019
p = 0.0310
Th1
Th2
Th17
i
4.380 ± 0.8184
2.543 ± 0.4213
1.993 ± 0.3746
Spleen
3.028 ± 0.5359
1.904 ± 0.2522
1.272 ± 0.1885
j
Spleen
k
Basophil
Eosinophil
Mast cell
CD49b
Siglec-F
FcεRIα
4.193 ± 0.6427*
3.635 ± 0.7890
0.1325 ± 0.0198
Lymph nodes
6.652 ± 0.2107
4.246 ± 1.136
0.2188 ± 0.0566
c-Kit
Frequence of Lin- (%)
p = 0.0051

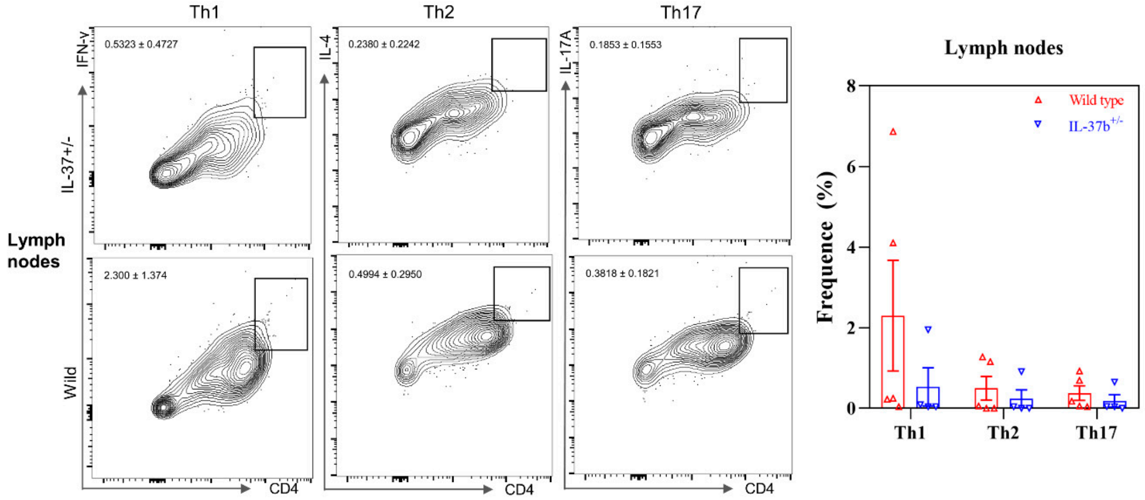

Figure 4. *In vivo* effects of the IL-37b$^{+/-}$ transgene on AD. (**a**) AD murine model schedule; (**b**) Ear swelling, skin moisture and scratching times; (**c**) Flow cytometry analysis of basophil, eosinophil and mast cell population in the ear tissue; (**d**) Quantitative analysis of result in Figure c; (**e**) Flow cytometry analysis of basophil, eosinophil and mast cell population in the spleen; (**f**) Quantitative analysis of result in Figure e; (**g**) Flow cytometry analysis of Th1, Th2 and Th17 cell population in the ear tissue; (**h**) Quantitative analysis of result in Figure g; (**i**) Flow cytometry analysis of Th1, Th2 and Th17 cell population in the spleen; (**j**) Quantitative analysis of result in Figure i. (**k**) Flow cytometry analysis and quantitative analysis of basophil, eosinophil and mast cell population in the lymph nodes; (**l**) Flow cytometry analysis and quantitative analysis of Th1, Th2 and Th17 cell population in the lymph nodes. Bar charts are shown as mean ± SEM of n= 4 or 5. *p < 0.05 and **p < 0.01 when compared between the groups.

a

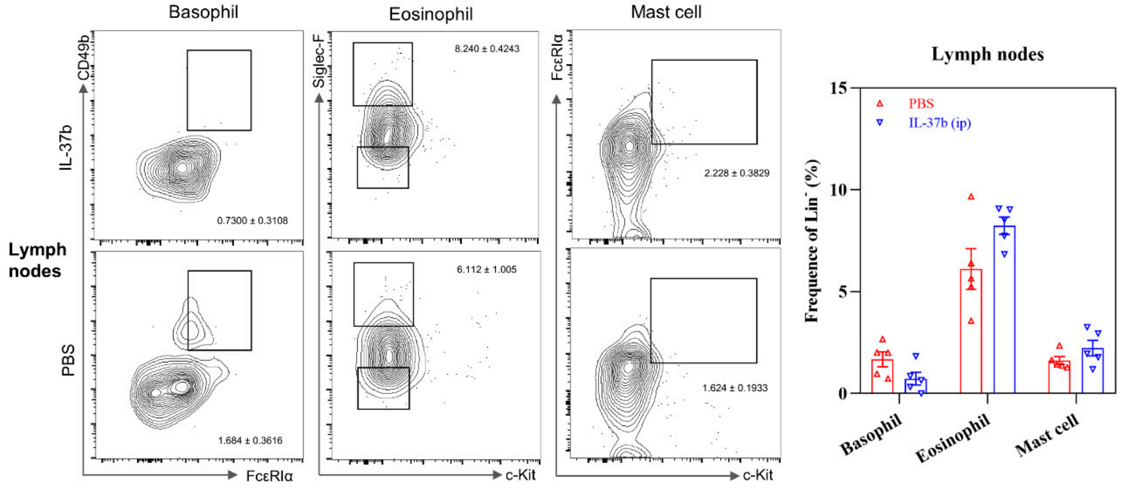

b

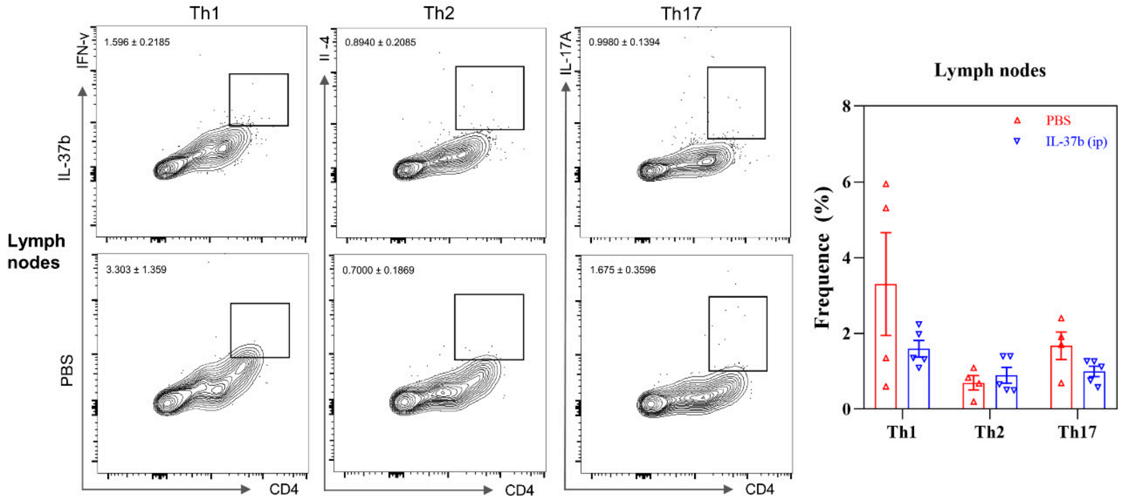

Figure 5. *In vivo* effects of the IL-37b injection on AD. (**a**) Flow cytometry analysis and quantitative analysis of basophil, eosinophil and mast cell population in the lymph nodes; (**b**) Flow cytometry analysis and quantitative analysis of Th1, Th2 and Th17 cell population in the lymph nodes. Bar charts are shown as mean ± SEM of n= 4 or 5.

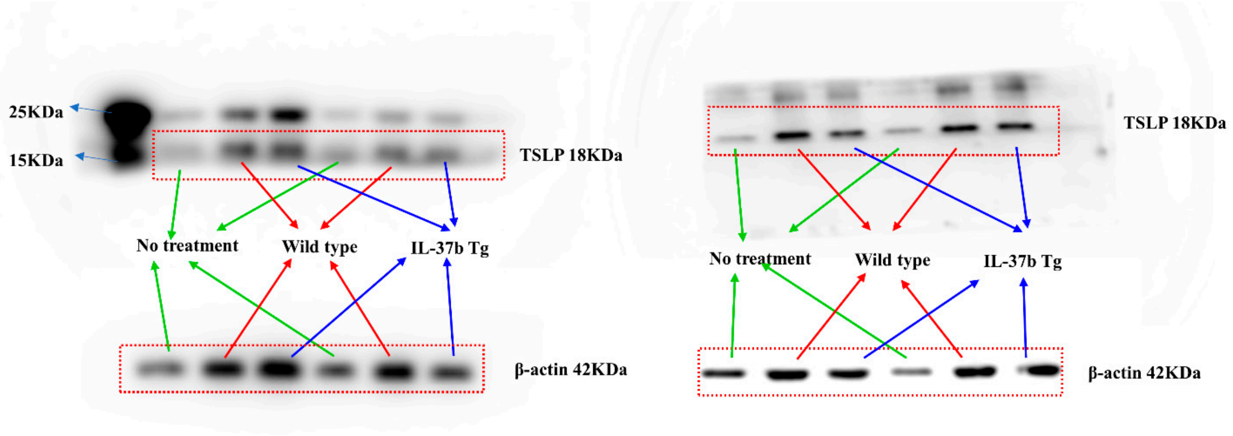

Figure 6. Raw Western blot results of Figure 8.

a

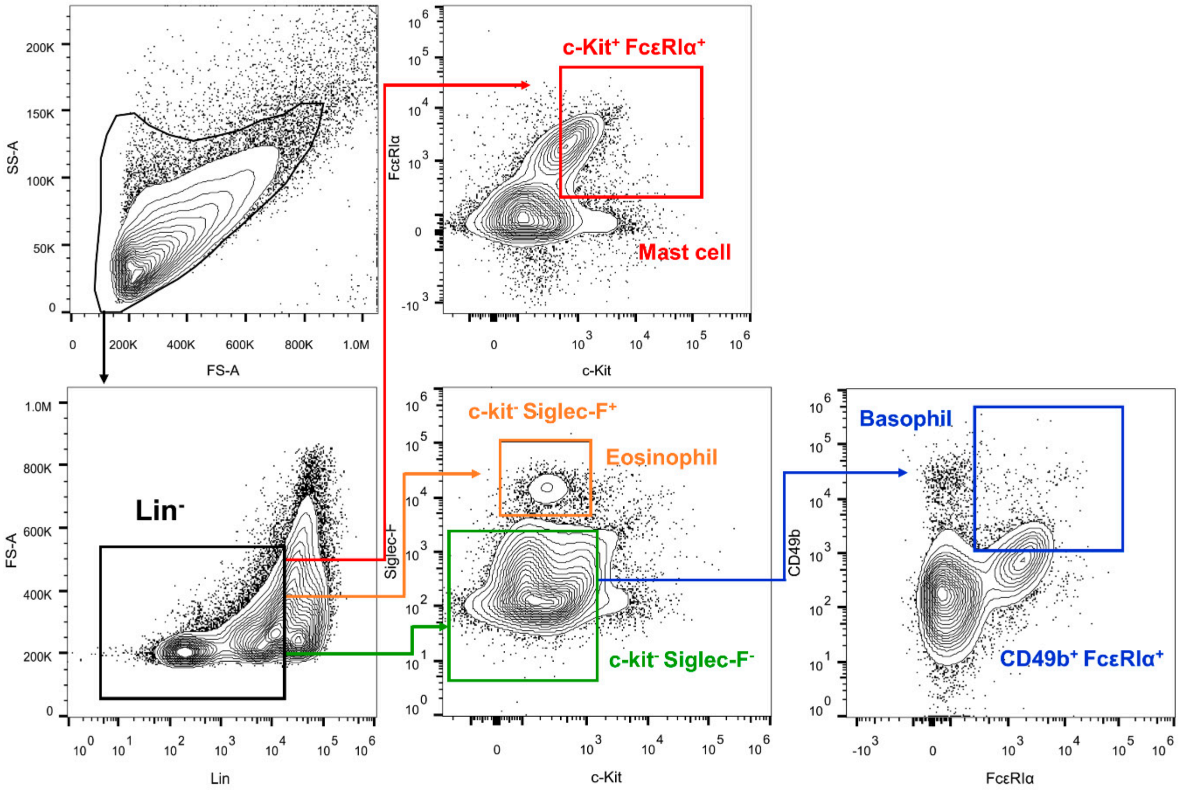

SS-A
FS-A
FcεRIα
c-Kit
c-Kit⁺ FcεRIα⁺
Mast cell
Lin⁻
Lin
FS-A
Siglec-F
c-kit⁻ Siglec-F⁺
Eosinophil
c-kit⁻ Siglec-F⁻
CD49b
Basophil
CD49b⁺ FcεRIα⁺
FcεRIα

b

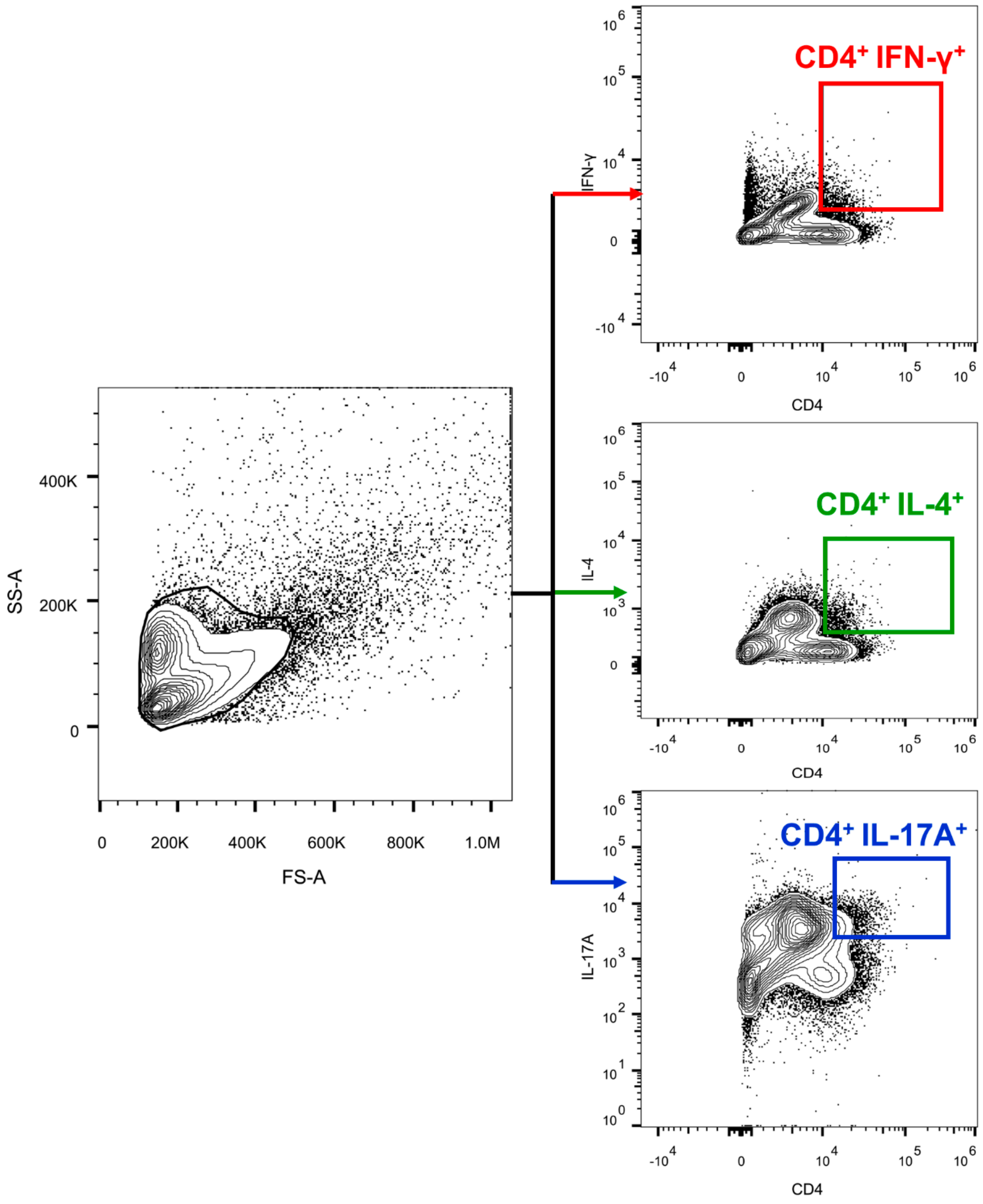

Figure 7. Flow cytometry gating strategies for (**a**) basophils, eosinophils and mast cells; and (**b**) Th1, Th2, and Th17 cells.

Table 1. Primers for human genes analyzed using real time PCR.

Gene		Primer Sequences (5’-3’)
IL-4	Forward	ACATTGTCACTGCAAATCGACACC
	Reverse	TGTCTGTTACGGTCAACTCGGTGC
CD63	Forward	ATGCAGGCAGATTTTAAGTGCT
	Reverse	GTTCTTCGACATGGAAGGGATTT
CD203c	Forward	AGATGCTAAATCTCACCCAAGA
	Reverse	GAGGGATGATAAAGGGTAGGAC
TNF-α	Forward	CCCAGGGACCTCTCTCTAATC
	Reverse	ATGGCTACAGGCTTGTCACT
IL-18Rα	Forward	CACAGACACCAAAAGCTTCATC
	Reverse	CACAGTCACTAGGCACACTAC
IL-1R8	Forward	TCAGTGGCTCTGAACTGCAC
	Reverse	GTACCAGAGCAGCACGTTGA
TSLP	Forward	GCCATGAAAACTAAGGCTGC
	Reverse	CGCCACAATCCTTGTAATTG
Smad3	Forward	CATCGAGCCCCAGAGCAATA
	Reverse	GTGGTTCATCTGGTGGTCACT
IL-37b	Forward	GCTCAGGTGGGCTCCTGGAA
	Reverse	GCTGACCTCACTGGGGCTCA
GAPDH	Forward	ATGGGGAAGGTGAAGGTCG
	Reverse	GGGGTCATTGATGGCAACAATA
